# Contribution of sortase SrtA2 to *Lactobacillus casei* BL23 inhibition of *Staphylococcus aureus* internalization into bovine mammary epithelial cells

**DOI:** 10.1371/journal.pone.0174060

**Published:** 2017-03-21

**Authors:** Renata F. S. Souza, Julien Jardin, Chantal Cauty, Lucie Rault, Damien S. Bouchard, Luis G. Bermúdez-Humarán, Philippe Langella, Vicente Monedero, Núbia Seyffert, Vasco Azevedo, Yves Le Loir, Sergine Even

**Affiliations:** 1 INRA, UMR 1253 STLO, Rennes, France; 2 Agrocampus Ouest, UMR1253 STLO, Rennes, France; 3 Universidade Federal de Minas Gerais (UFMG), Belo Horizonte, Brazil; 4 Micalis Institute, INRA, AgroParisTech, Paris-Saclay University, Jouy-en-Josas, France; 5 Instituto de Agroquímica y Tecnología de Alimentos-CSIC, Paterna, Spain; The University of Tokyo, JAPAN

## Abstract

Probiotics have been considered as a promising strategy to prevent various diseases in both humans and animals. This approach has gained interest in recent years as a potential means to control bovine mastitis. In a previous study, we found that several *L*. *casei* strains, including BL23, were able to inhibit the internalization of *S*. *aureus*, a major etiologic agent of mastitis, into bovine mammary epithelial cells (bMEC). This antagonism required a direct contact between *L*. *casei* and bMEC or *S*. *aureus*, suggesting the inhibition relied on interactions between *L*. *casei* cell surface components and bMEC. In this study, we have investigated the impact of some candidates which likely influence bacteria host cell interactions. We have shown that *L*. *casei* BL23 *fbpA* retained its inhibitory potential, indicating that *L*. *casei* BL23 antagonism did not rely (solely) on competition between *S*. *aureus* and *L*. *casei* fibronectin-binding proteins for adhesion to bMEC. We have then investigated the impact of four sortase mutants, *srtA1*, *srtA2*, *srtC1* and *srtC2*, and a double mutant (*srtA1-srtA2*) on *L*. *casei* BL23 inhibitory potential. Sortases are responsible for the anchoring on the bacterial cell wall of LPXTG-proteins, which reportedly play an important role in bacteria-host cell interaction. All the *srt* mutants tested presented a reduced inhibition capacity, the most pronounced effect being observed with the *srtA2* mutant. A lower internalization capacity of *L*. *casei srtA2* into bMEC was also observed. This was associated with several changes at the surface of *L*. *casei* BL23 *srtA2* compared to the wild type (wt) strain, including altered abundance of some LPXTG- and moonlighting proteins, and modifications of cell wall structure. These results strongly support the role of sortase A2 in *L*. *casei* BL23 inhibition against *S*. *aureus* internalization. Deciphering the contribution of the cell surface components altered in *srtA2* strain in the inhibition will require further investigation.

## Introduction

The increasing amount of data on the relationship between host health and microbiota composition has raised a real interest in the development of probiotic solutions for human and animal health, considering that these probiotics could restore a balanced microbiota and, consequently, ecosystem homeostasis. They can also exert or contribute to a barrier effect with regard to pathogen colonization and its associated symptoms [1–3]. In this context, lactic acid bacteria (LAB) are candidates of choice due to their GRAS status (Generally Recognized as Safe) and to the well-documented beneficial effects they exert on intestinal and vaginal human ecosystems [4–6].

Probiotic solutions have been considered as a promising strategy for the control of various diseases in humans [7, 8] and in animals [9] as well. They are of special interest in animal health and are regarded as an alternative means to reduce massive antibiotic use against infectious diseases, including ruminant mastitis in dairy farms [9–13]. *Staphylococcus aureus*, a Gram-positive opportunistic pathogen, is one of the main pathogens involved in mastitis, responsible for great economic losses [14, 15].

To explore the probiotic potential of LAB in a mastitis context, we recently tested the ability of three *Lactobacillus casei* strains, including the well-characterized probiotic strain BL23, to compete with *S*. *aureus* for bovine mammary epithelial cell (bMEC) adhesion and internalization [16]. In this study, we found that *L*. *casei* antagonism with regard to *S*. *aureus* internalization into bMEC required a direct contact between *L*. *casei* and bMEC or *S*. *aureus*. We thus postulated that internalization inhibition relied on interactions between *L*. *casei* cell surface components and bMEC.

This study aimed at demonstrating the involvement of cell surface components in the inhibition capacities of *L*. *casei* with regard to *S*. *aureus* internalization and at identifying some candidates that might be involved in this phenomenon. Many cell surface components are known to interact with host cells in *L*. *casei*. It would therefore be impossible to comprehensively investigate each one of them. One of the main mechanisms involved in *S*. *aureus* internalization relies on the interaction between *S*. *aureus* fibronectin-binding protein and integrin α5 β1 via fibronectin bridging [17, 18]. *L*. *casei* produces a fibronectin binding protein (FbpA). We thus first postulated that inhibition could rely on a competition for fibronectin attachment through the interaction between *L*. *casei* FbpA and fibronectin. To test this hypothesis, we evaluated the inhibition capacity of *L*. *casei* BL23 *fbpA* [19]. Secondly, we evaluated the inhibition capacities of *L*. *casei* BL23 sortase mutants [20]. These enzymes are involved in the processing of cell wall-anchored (CWA) proteins. Sortases recognize the LPXTG motif characteristic of CWA proteins, cleave and covalently bind the mature moiety of the protein to the peptidoglycan. Four genes encoding sortases have been identified in *L*. *casei* BL23, *srtA1*, *srtA2*, *srtC1* and *srtC2*. They potentially control the anchoring of 23 proteins that harbor the LPXTG motif in their C-terminal region. Eight of these proteins exhibit adhesion-related functions. Furthermore, the *srtA1-srtA2* double mutant and the *srtA2* mutant show a reduced ability of adhesion to Caco-2 cells [20]. We thus hypothesized that sortases and their substrates could contribute to the inhibition capacities of *L*. *casei* against bMEC colonization by *S*. *aureus*. The impact of each single sortase mutant and the *srtA1-srtA2* double mutant on the inhibition of *S*. *aureus* colonization and on the colonization capacities of *L*. *casei* BL23 on bMEC was explored. The impact of sortase mutations on the cell surface was monitored through analysis of the cell surface proteome and cell shape, revealing several bacterial surface components that could contribute to the inhibitory potential of *L*. *casei*.

## Material and methods

### Bacterial strains and culture conditions

*L*. *casei* BL23 wild type and the previously constructed mutants are listed in S1 Table [19–22]. *S*. *aureus* Newbould 305 (hereafter referred to as N305), a well-characterized strain of *S*. *aureus* isolated from bovine mastitis [23], was used for inhibition tests.

For *S*. *aureus*, subculture was carried out in brain-heart infusion medium (BHI; pH 7.4; BD, Le Pont de Claix, France) at 37°C under agitation (180 rpm), and *L*. *casei* strains were sub-cultured in Man Rogosa Sharpe medium (MRS; pH 6.8; BD) at 37°C without shaking. Subcultures were washed once with phosphate-buffered saline (PBS) and suspended at different concentrations in Dulbecco’s modified Eagle’s medium (DMEM; pH 7.4; D. Dutscher, Brumath, France) for invasion assays, or in ultra-filtered (UF) milk medium for enzymatic shaving of surface experiments. Alternatively, subcultures for invasion assays were performed on ultra-filtered milk medium so as to be in agreement with conditions used for enzymatic shaving of surface experiments. Whenever necessary (i.e., for *L*. *casei* BL23 mutant strains), erythromycin (Sigma Aldrich, Saint Louis, USA) was added to the culture at a final concentration of 5 μg/mL.

Bacterial concentrations in subcultures were estimated by spectrophotometric measurements at 600 nm with a VWR V-1200 spectrophotometer. They were further confirmed by determination of the bacterial population using a micromethod, as previously described [24]. The *S*. *aureus* population (in CFU/mL) was determined on mannitol salt agar (MSA; D. Dutscher) after 24 h of incubation at 37°C. The *L*. *casei* population was determined on MRS (pH 5.4) and incubated anaerobically for 48 h at 30°C in an anaerobic jar.

### Mammary epithelial cell culture

The bovine mammary epithelial cell line (bMEC) MAC-T (Nexia Biotechnologies, Quebec, Canada) was cultured in DMEM containing 10% fetal calf serum (FCS) (D. Dutscher), 100 U/mL penicillin (Ozyme, Montigny-le-Bretonneux, France), 10 mg/mL streptomycin (Ozyme) and 5 μg/mL insulin (Sigma Aldrich) in T75 cell culture flasks (Starlab, Orsay, France). Cells were incubated at 37°C in a humidified incubator with 5% carbon dioxide (CO_2_). They were cultured to a confluent monolayer, treated with 0.05% trypsin (Sigma Aldrich) and resuspended in fresh DMEM to obtain a final concentration of 2 x 10^5^ cells/mL.

Adhesion and internalization assays were performed in 12-well plates with 2 x 10^5^ cells seeded per well. Cells were incubated in a humidified incubator with 5% CO_2_ at 37°C overnight to obtain a confluent monolayer.

### Internalization assays

Internalization assays were performed according to Bouchard et al. (2013) [16]. Confluent monolayers of MAC-T cells (at 2.5 x 10^5^ cells/well) were washed twice with phosphate-buffered saline and incubated for 2 hours at 37°C and 5% CO_2_ with 1 mL of *L*. *casei* BL23 wt or mutant strains resuspended in DMEM, with a multiplicity of infection (MOI) of 400:1 or 2,000:1. For internalization inhibition assays, *S*. *aureus* N305 (MOI 100:1) and *L*. *casei* strains were co-incubated with cells for two hours. Cells were then washed four times with PBS and incubated with DMEM containing 100 μg/mL gentamicin (Pan Biotech, Aidenbach, Germany) for two additional hours in order to kill adhered extracellular bacteria. The MAC-T cell monolayer was then washed four times with PBS, treated with trypsin, centrifuged for 5 minutes at 800 x g and lysed with triton at 0.01%. Internalized bacterial populations were determined using a micromethod (see above). The *L*. *casei* population was determined following 48 h incubation at 30°C on MRS (pH 5.4) in an anaerobic jar. The *S*. *aureus* population was determined on MSA after 24 h of incubation at 37°C.

For internalization inhibition assays, internalization of *S*. *aureus* was used as a reference. The inhibition rate of internalization was defined as the internalized *S*. *aureus* population in the presence of *L*. *casei* with respect to the internalized *S*. *aureus* population in the reference experiment.

### Enzymatic shaving of surface proteins

Enzymatic shaving of *L*. *casei* was adapted from Le Marechal et al. (2015) [25]. *L*. *casei* was cultured in UF milk medium [26] until the stationary growth phase (48 h). This medium was retained for technical reasons since cultures of *L*. *casei* on MRS led to contaminating signals in nano-LC-MS analysis in relation to the presence of Tween 20. Moreover, UF milk medium contains fewer proteins, which resulted, once again, in fewer “contaminating signals”. The bacteria were centrifuged (8,000 x g, 10 min, 4°C), washed three times in one volume of PBS (pH 8.5) containing 5 mM dithiothreitol (DTT) (Sigma Aldrich) and resuspended in 0.5 mL of the same buffer. The 0.5 mL suspension containing 2 x 10^10^ bacteria was treated with 4 mg/mL Sequencing Grade Modified Trypsin (V5111, Promega, Madison, USA) for 1 hour at 37°C with gentle agitation (180 rpm) (“shaving”). Bacteria were then removed by centrifugation (8,000 x g, 10 minutes, 4°C) and the supernatant was filtered (0.2 μm filter, Nalgene, Rochester, USA). The viability of *L*. *casei* was checked after shaving using a micromethod. For a complete digestion of the peptides released by shaving, the supernatant was further treated with 1 μg of trypsin for 16 hours at 37°C and 120 rpm. Trypsin digestion was stopped by adding trifluoroacetic acid (TFA) to a final concentration of 0.15% (v/v), and the supernatants containing peptides were then concentrated in a Speed-Vac concentrator prior to nano-LC–MS/MS analysis. Shaving was performed in triplicate on three independent cultures.

### Nano LC-MS analyses

Experiments were performed using a nano RSLC Dionex U3000 system fitted to a Q-Exactive mass spectrometer (Thermo Scientific, San Jose, USA) equipped with a nano- electrospray ion source. A preliminary sample concentration step was performed on a C18 pepMap100 reverse-phase column (C18 column: inner diameter (i.d.): 300 μm; length: 5 mm; particle size: 5 μm; pore size: 100 Å; Dionex, Amsterdam, the Netherlands). Peptide separation was performed on a reversed-phase column (PepMap RSLC C18: i.d.: 75 μm; length: 150 mm; particle size: 3 μm; pore size: 100 Å; Dionex), with a column temperature of 35°C, using solvent A (2% (v/v) acetonitrile, 0.08% (v/v) formic acid and 0.01% (v/v) TFA in deionized water) and solvent B (95% (v/v) acetonitrile, 0.08% (v/v) formic acid and 0.01% (v/v) TFA in deionized water). Peptides were separated using a gradient of 5 to 35% of solvent B over 70 min, followed by 35 to 85% of solvent B over 5 min at a flow rate of 0.3 μL/min. Eluted peptides were directly electrosprayed into the mass spectrometer operating in positive ion mode with a voltage of 2 kV. Spectra were recorded in full MS mode and selected in a mass range 230-2000 m/z for MS spectra with a resolution of 70,000 at m/z 200. For each scan, the ten most intense ions were selected for fragmentation. MS/MS spectra were recorded with a resolution of 17,500 at m/z 200, and the parent ion was subsequently excluded from MS/MS fragmentation for 20 s. The instrument was externally calibrated according to the supplier's instructions.

Peptides were identified from the MS/MS spectra using X!Tandem pipeline software (Plateforme d'Analyse Protéomique de Paris Sud-Ouest (PAPPSO), INRA, Jouy-en-Josas, France, http://pappso.inra.fr) [27]. The search was performed against a database composed of (i) a portion of the UniProtKB database corresponding to *L*. *casei* BL23 (http://www.uniprot.org/taxonomy/543734), and (ii) an in-house database composed of major milk and egg proteins taken from www.uniprot.org (207 proteins in total). The latter was used to remove signals associated with milk proteins since cultures were performed on milk-derived medium. Database search parameters were specified as follows: trypsin cleavage was used and the peptide mass tolerance was set to 10 ppm for MS and 0.05 Da for MS/MS. Oxidation of methionine and phosphorylation on threonine, serine and tryptophan were selected as a variable modification. For each peptide identified, a minimum score corresponding to an e-value below 0.05 was considered as a prerequisite for peptide validation.

### Transmission electron microscopy

*L*. *casei* was grown in UF milk medium until the stationary phase of growth so as to be in accordance with growth conditions used for enzymatic shaving of surface experiments. Bacteria were recovered by centrifugation (6,000 x g, 5 min) and fixed for three hours with gentle agitation (150 rpm) in 0.1 M sodium cacodylate buffer (pH 7.2) containing 2.5% glutaraldehyde. Bacteria were then washed four times and stored at 4°C overnight in a cacodylate buffer. Post-fixation of the bacteria was performed in a cacodylate buffer containing 1% osmium tetroxide (Electron Microscopy Sciences, Hatfield, USA) for 1 hour, and bacteria were then embedded in 1.5% agar (Biokar Diagnostics, Beauvais Cedex, France). Progressive dehydration was performed with ethanol at concentrations of 50-100% and cells were progressively embedded in Epon (Electron Microscopy Sciences).

Transmission electron microscopy was performed at the MRic platform (University of Rennes 1, Rennes, France). Thin sections of 90 nm were prepared with diamond knives on an Ultracut, placed on cupper grids, stained with 2% uranyl acetate, and subsequently analyzed with JEOL 1400 TEM (Jeol, Tokyo, Japan) operating at 120 kv accelerating voltage.

Digital images were acquired using the Gatan SC1000 Orius® CCDcamera (4008 x 2672), set up with Gatan Digital Micrograph™ imaging software (Gatan, Pleasanton, USA). TEM images were performed at the Microscopy Rennes Imaging Center platform (MRic-MET, University of Rennes 1, Rennes, France). The same procedure was used to visualize *L*. *casei* and/or *S*. *aureus* internalized into bMEC, starting from 2.5 x 10^5^ cells. In addition to the presence of internalized bacteria in infected MAC-T cells compared to non-infected cells, degradation vesicles, as defined as vesicles containing degraded bacteria, were observed.

Image analysis was performed using ImageJ, version 1 [28]. Two measurements of the bacterial wall thickness were made, with a total of ten measurements in each of the three biological replicates.

### Assessment of oxidative stress resistance

Resistance of *L*. *casei* wt and mutant strains to oxidative stress was evaluated through exposure to hydrogen peroxide (H_2_O_2_). Strains were grown on MRS and were harvested in the exponential phase (OD600 nm: ~ 0.8) or stationary phase (24 h) of growth. H_2_O_2_ (30% w/v, Sigma Aldrich) was added to 14 mL of culture to a final concentration of 0.1% and 0.2% for the exponential phase and 0.25% and 0.5% for the stationary phase, and incubated at 37°C without agitation. Samples were collected at time 0 (without addition of H_2_O_2_), and at 10, 20 and 30 minutes of exposure in order to evaluate the surviving *L*. *casei* population. H_2_O_2_ was eliminated by adding catalase (Sigma Aldrich) at a final concentration of 10 U/mL and the *L*. *casei* population was determined by plating serial dilution on MRS and incubating plates for 48 h at 37°C.

### Analysis of the auto-aggregation capability

The auto-aggregation test was performed according to Ocaña and Nader-Macias (2002) [29] with some modifications. *L*. *casei* was cultured in UF milk medium for 48 hours at 37°C and the percentage of auto-aggregation was calculated as follows:

$$Auto-aggregation\;(\%)=\left(1-\frac{{OD}_{suspension}}{{OD}_{total}}\right)x100$$

where OD_suspension_ is the optical density (OD) of the cell suspension at 600 nm after 48 hours of *L*. *casei* growth under static conditions, and OD_total_ is the cell suspension OD measured on the same culture after homogenization. The total bacterial population was also determined by CFU counting to see if differences in OD were related to differences in auto-aggregation or growth capacities in these conditions.

### Statistical analysis

Statistical analysis of the bacterial surface proteome was performed as follows: every peptide identified by tandem mass spectrometry after enzymatic shaving of surface proteins was quantified using free MassChroQ software [30] before data treatment and statistical analysis within R software (R 3.2.2, Project for statistical computing). A specific R package called 'MassChroqR' was used to automatically filter dubious peptides for which the standard deviation of retention time was greater than 40 s and to regroup peptide quantification data into proteins.

Three different and complementary methods of analysis were used based on peak counting, spectral counting and XIC (Extracted Ion Chromatogram).

For peak counting and spectral counting analysis, a non-parametric Kruskall-Wallis H test was performed on proteins. A minimum difference of two quantified peaks between strains was retained for peak counting. Proteins with a p-value < 0.05 were considered to be significantly different.

For XIC-based quantification, normalization was performed to take possible global quantitative variations between LC-MS runs into account. For each LC-MS run, the ratio of all peptide values to their value in the chosen reference run was computed. Normalization was performed by dividing peptide values by the median value of peptide ratios. Peptides shared between different proteins were automatically excluded from the dataset, as were peptides present in less than 80% of the samples. Missing data were then imputed from a linear regression based on other peptide intensities for the same protein [31]. Analysis of variance was used to determine proteins with significantly different abundances between mutant strains.

Other statistical analyses were performed with GraphPad Prism software, version 5.01 [32]. Differences between groups were assessed by one-way ANOVA, followed by Bonferroni's Multiple Comparison Test and Student’s t-test, considering a P value of less than 0.05. Each experiment was conducted in biological triplicate.

## Results

### Inhibition of *S*. *aureus* internalization into bMEC by *L*. *casei* mutant strains

**Fig 1:**
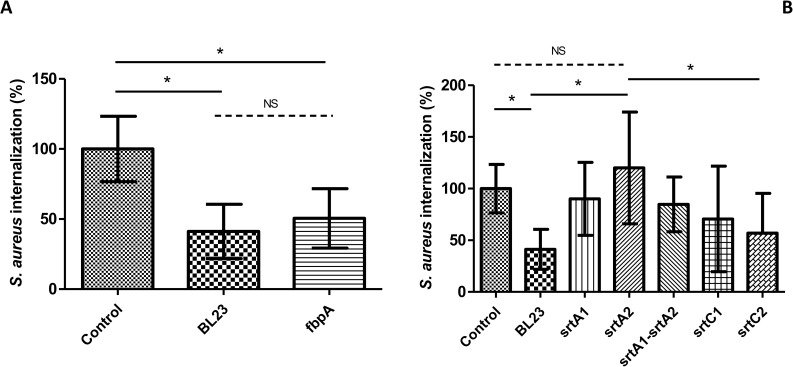
Inhibition of *S*. *aureus* internalization into bMEC by *L*. *casei*. Internalization rates of *S*. *aureus* N305 after 2 h of interaction with bMEC and co-incubation with wt, *fbpA* (panel A) and sortase mutant strains (panel B) of *L*. *casei* BL23 at an MOI of 2,000:1. *S*. *aureus* was used at an MOI of 100:1. The internalization assay of *S*. *aureus* alone was used as a reference (control). Internalization rates were then defined as the internalized *S*. *aureus* population in the presence of the different *L*. *casei* strains relative to the internalized *S*. *aureus* population of the reference experiment. Data are presented as means ± standard deviations. Each experiment was done in triplicate, and differences between groups were compared using one-way ANOVA with Bonferroni's Multiple Comparison Test. *: P < 0.05.

The inhibitory potential of *L*. *casei* BL23 wt and *fbpA* and *srt* mutant strains against *S*. *aureus* N305 internalization into bMEC was evaluated using MOIs of 400:1 and 2,000:1 for *L*. *casei* and 100:1 for *S*. *aureus*. No significant difference in the *S*. *aureus* internalization rate was observed in the presence of *L*. *casei* BL23 wt and mutant strains at an MOI of 400:1 (data not shown). A significant reduction of 58% of the internalization rate of *S*. *aureus* into bMEC was observed in the presence of *L*. *casei* BL23 wt at an MOI of 2,000:1. A similar reduction of the internalization rate of *S*. *aureus* into bMEC was observed in the presence of *L*. *casei* BL23 *fbpA* (Fig 1, panel A). On the contrary none of the sortase mutants showed a significant inhibition of *S*. *aureus* internalization (Fig 1, panel B). A significant and maximum release of inhibition was observed with the *srtA2* mutant strain when compared to the inhibition observed with the control *L*. *casei* BL23 wt. In order to confirm that the release of inhibition was due to *srtA2* inactivation and not related to a secondary mutation, a second *srtA2* mutant strain, named *ΔsrtA2* and obtained by double crossing over (S1 Table), was tested. A significant release of inhibition was also observed with this second srtA2 mutant strain compared to *L*. *casei* BL23 wt strain as illustrated by an *S*. *aureus* internalization rate of 86% +/-26% relative to the control in the presence of *L*. *casei ΔsrtA2*.

**Fig 2:**
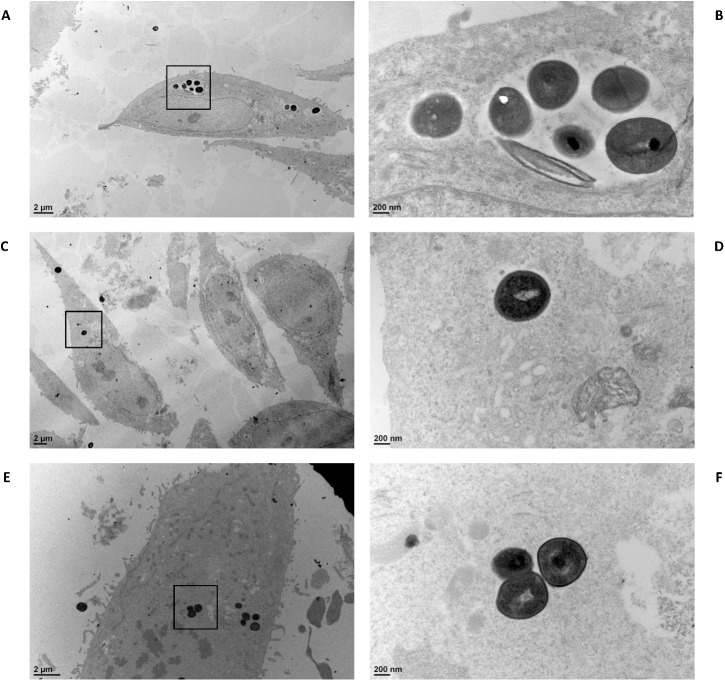
Microscopic observation of internalized *S*. *aureus*. Internalization of *S*. *aureus* N305 as observed by transmission electron microscopy. *S*. *aureus* N305 (at an MOI of 100:1) was incubated for 2 h with bMEC either alone (A, B) or in the presence of *L*. *casei* BL23 wt (C, D) or *srtA2* mutant (E, F) strains, at an MOI of 2,000:1.

In order to be in accordance with enzymatic shaving experiments that were done using UF-medium grown cultures for technical reasons (see Material and Methods for details), we further confirmed that similar results were obtained when *L*. *casei* subcultures were performed on UF-medium instead of MRS prior to invasion assays (data not shown). Results were further confirmed by transmission electron microscopy (TEM) observation (Fig 2). A greater number of internalized *S*. *aureus* was observed when cells were infected by *S*. *aureus* alone (Fig 2, panels A and B) and in the presence of *L*. *casei srtA2* (Fig 2, panels E and F), compared to internalized *S*. *aureus* in the presence of the *L*. *casei* wt strain (Fig 2, panels C and D).

### Reduced internalization of *L*. *casei srtA2* into bMEC

**Fig 3:**
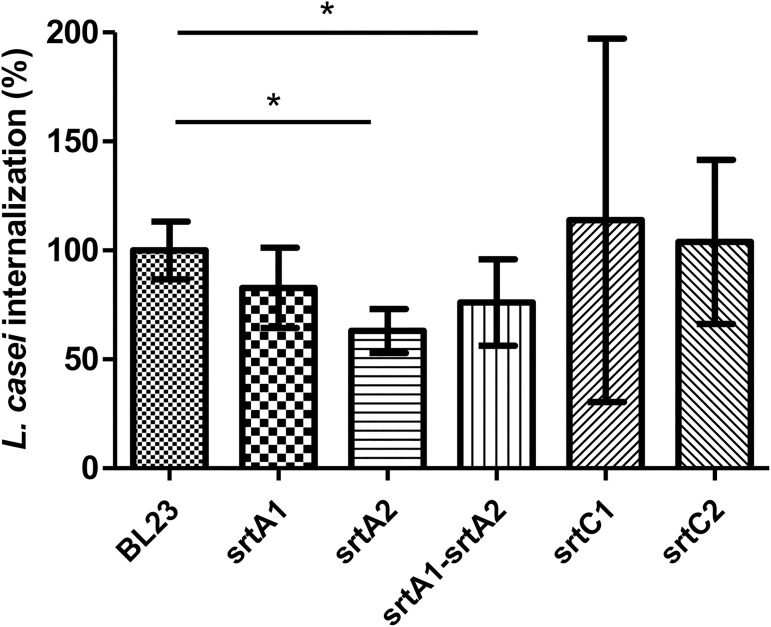
Internalization of wild type and mutant strains of *L*. *casei* BL23 into bMEC. *L*. *casei* populations internalized into bMEC were determined after 2 h of interaction at an MOI 2,000:1. The internalization assay of the *L*. *casei* BL23 wild type (wt) strain was used as a reference. Internalization rates were defined as the internalized population of mutant strains relative to the internalized *L*. *casei* BL23 wt strain population. Data are presented as mean ± standard deviations. Each experiment was done in triplicate and differences between groups were compared using Student’s t-test. *: P < 0.05.

**Fig 4:**
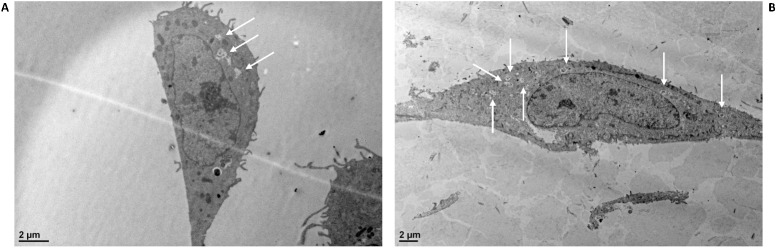
**Internalization of *L*. *casei* BL23 wt (A) and *srtA2* mutant (B) strains as observed by transmission electron microscopy**. Degradation vesicles (white arrows) were observed in a greater proportion in cells containing mutant *srtA2*.

Adhesion and internalization capacities of *L*. *casei* BL23 wt and sortase mutant strains were determined on bMEC with an MOI of 400:1 and 2,000:1. No significant difference in the adhesion rate was observed in sortase mutant strains compared to the BL23 wt strain (data not shown). However, a significant reduction of internalization rates was obtained for *srtA2* (37%) and *srtA2-srtA1* (24%) strains compared to BL23 wt with an MOI 2,000: 1 (Fig 3). Of note, TEM observation of bMEC infected with BL23 wt or *srtA2* showed degradation vesicles in greater abundance in the bMEC infected with the *srtA2* mutant strain (Fig 4). The observed reduction in the *srtA2* mutant internalization may thus result either from a lower capability of internalization into bMEC and or from a lower survival rate of this strain once internalized into the bMEC.

### *L*. *casei srtA2* is less resistant to oxidative stress

**Fig 5:**
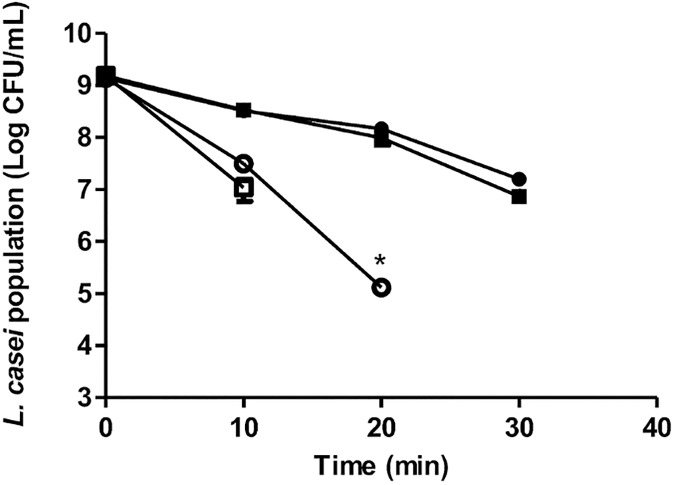
Resistance of *L*. *casei* BL23 wt and *srtA2* strains to H_2_O_2_. Resistance of *L*. *casei* BL23 wt (●, ○) and *srtA2* (■, □) strains to H_2_O_2_ was evaluated in the stationary phase of growth of *L*. *casei* (24h—MRS). The residual population was evaluated at 0, 10, 20 and 30 min after exposure to 0.25% (●, ■) and 0.5% H_2_O_2_ (○, □). Data are presented as means ± standard deviations. Each experiment was done in triplicate, and differences between groups were compared using Student’s t-test. *: P < 0.05.

Oxidative stress is a major stress encountered by bacteria once they have been internalized into eukaryotic cells. Resistance to oxidative stress was evaluated by measuring the resistance of *L*. *casei* BL23 wt and *srtA2* strains to H_2_O_2_ exposure in the exponential and stationary phases of growth. No significant difference in survival to H_2_O_2_ exposure was observed between strains when tested in the exponential phase (data not shown). In contrast, when tested in the stationary phase, *L*. *casei* BL23 *srtA2* was more sensitive to H_2_O_2_ exposure than BL23 wt (Fig 5). The difference was more pronounced after 20 minutes of exposure to 0.5% H_2_O_2_, as illustrated by a survival rate of approximately 1 x 10^5^ CFU/mL of *L*. *casei* BL23 wt, whereas there was no residual population of the *srtA2* strain.

### Altered profile of surface proteins in *L*. *casei srtA2*

The impact of *srtA2* disruption on the cell surface proteome of *L*. *casei* BL23 was determined by enzymatic shaving. The absence of cell lysis during trypsin treatment was confirmed by measuring *L*. *casei* population in the cell lysis buffer before and after shaving. The total population was 2.7 x 10^10^ CFU/mL before trypsin treatment for both BL23 wt and *srtA2* mutant strain. Trypsin treatment did not change significantly *L*. *casei* population. Hence, following 1h of incubation in cell lysis buffer, *L*. *casei* BL23 wt population was 3.20 x 10^10^ CFU/mL and 3.18 x 10^10^ CFU/mL in the presence and absence of trypsin respectively. Likewise, *L*. *casei srtA2* population was 3.05 x 10^10^ CFU/mL and 3.28 x 10^10^ CFU/mL in the presence and absence of trypsin respectively. Data analysis was performed using three different and complementary methods of analysis, based on peak counting, spectral counting and XIC (Extracted Ion Chromatogram). While the two former methods are based on the detection of spectra and thus reveal proteins that exhibit strong differences between conditions, the latter reveals proteins that are present in both conditions but with differential abundance. Complete datasets are presented in S2, S3 and S4 Tables. Proteomic data analysis revealed several changes in the cell surface proteome of BL23 *srtA2*. Hence, 57, 144 and 196 proteins were differentially abundant at the cell surface of *L*. *casei* BL23 *srtA2* compared to the BL23 wt strain using the peak counting, spectral counting and XIC methods, respectively. A selection of proteins differentially abundant at the cell surface between wt and *srtA2* mutant strains and discussed in this work is presented in Table 1. They include the proteins containing the LPXTG motif, those presumably located at the cell surface or involved in cell surface component biosynthesis, or those known to be involved in the interaction with the host, including moonlighting proteins and proteins involved in cellular stress response.

**Table 1:** Cell surface proteome of *L*. *casei* BL23 wt and *srtA2* mutant as determined by enzymatic shaving.

**Protein ID**	**Gene ID**	**Protein name**	**Gene name**	**Localization**	**Moonlighting**^**a**^	**Ratio *srtA2*/wt**^**b**^			**Function**
						**Peak counting**	**Spectral counting**	**XIC**	
**LPXTG motif containing proteins**									
B3W8P4	LCABL_02860	Beta-N-acetylglucosaminidase	*bnaG*	PSE	-			0.50	Physiological processes
B3WA51	LCABL_25040	Internalin-J	*inlJ*	PSE	-			2.35	Unknown
**Cell wall/membrane/envelope biogenesis**									
B3WF06	LCABL_18780	Bifunctional dimerization/transpeptidase penicillin-binding protein 2B	*pbp2B2*	Secreted	-		3.75	6.27	Peptidoglycan synthesis
B3W8K4	LCABL_02120	UDP-N-acetylmuramyl-tripeptide synthetase	*murE*	Cytoplasmic	-			1.47	Peptidoglycan synthesis
B3WCW2	LCABL_11280	N-acetylmuramoyl-L-alanine amidase	*lys*	Cytoplasmic	Yes		0.00^c^		Cell wall degradation
B3W9B1	LCABL_22130	Tyrosine-protein phosphatase	*wzb*	Cytoplasmic			0.32		Exopolysaccharide biosynthesis
B3W9B2	LCABL_22140	Cell envelope-related transcriptional attenuator	*wzr*	Secreted			- ^d^		Exopolysaccharide biosynthesis
B3W978	LCABL_22210	dTDP-glucose 4,6-dehydratase	*rmlB*	Cytoplasmic				3.83	Exopolysaccharide biosynthesis
B3W979	LCABL_22220	dTDP-4-dehydrorhamnose 3,5-epimerase	*rmlC*	Cytoplasmic				0.70	Exopolysaccharide biosynthesis
B3W9D2	LCABL_22340	exopolysaccharide biosynthesis protein	*wze*	Cytoplasmic				0.68	Exopolysaccharide biosynthesis
B3W9D3	LCABL_22350	Capsular polysaccharide biosynthesis protein	*wzd*	PSE				0.46	Exopolysaccharide biosynthesis
**Cell division**									
B3WDV7	LCABL_14770	Septation ring formation regulator ezrA	*ezrA*	PSE	-			2.60	Cell division
B3WEU8	LCABL_18190	Signal recognition particle-docking protein	*ftsY*	Cytoplasmic	-	1.23			Cell division
B3WDY9	LCABL_15090	Cell-division initiation protein (Septum placement)	*divIVA*	Cytoplasmic	-			0.39	Cell division
B3WCN1	LCABL_10450	Cell division protein FtsX	*ftsX*	Cytoplasmic	-		0.21		Cell division
**Stress response**									
B3WCP7	LCABL_10620	Thioredoxin reductase	*trxB2*	Cytoplasmic	-	1.13		2.65	Oxidative stress response
**Protein ID**	**Gene ID**	**Protein name**	**Gene name**	**Localization**	**Moonlighting**^**a**^	**Ratio *srtA2*/wt**^**b**^			**Function**
						**Peak counting**	**Spectral counting**	**XIC**	
B3WC30	LCABL_08080	Thiol peroxidase (Hydroperoxide reductase, Peroxiredoxin)	*tpx*	Cytoplasmic	-			1.36	Oxidative stress response
B3WCJ1	LCABL_10060	Glutathione peroxidase	*bsaA*	Cytoplasmic	-			0.47	Oxidative stress response
B3WE57	LCABL_15770	Chaperone ClpB	*clpB*	Cytoplasmic	-		1.72		Heat shock response
B3WAM9	LCABL_26830	ATP-dependent Clp protease, ATP-binding subunit ClpC	*clpC*	Cytoplasmic	-	1.25	1.89		Heat shock response
B3WCR2	LCABL_10770	ATP-dependent Clp protease proteolytic subunit	*clpP*	Cytoplasmic	-	1.58	2.22		Heat shock response
B3WEQ8	LCABL_17790	Protein GrpE	*grpE*	Cytoplasmic	-			0.70	Heat shock response
B3W9A4	LCABL_22060	1,4-alpha-glucan branching enzyme (glycogen branching enzyme)	*glgB*	Cytoplasmic	-			0.64	Carbohydrate metabolism
B3W9A3	LCABL_22050	Glucose-1-phosphate adenylyltransferase	*glgC*	Cytoplasmic	-			0.70	Carbohydrate metabolism
B3W9A0	LCABL_22020	Glycogen phosphorylase	*glgP*	Cytoplasmic	-	0.71	0.25		Carbohydrate metabolism
**Moonlighting proteins**									
B3WCW4	LCABL_11300	Glyceraldehyde 3-phosphate dehydrogenase	*gap-1*	Cytoplasmic	Yes			0.44	Carbohydrate metabolism
B3WE98	LCABL_16180	Glyceraldehyde 3-phosphate dehydrogenase	*gapB*	Cytoplasmic	Yes	0.63			Carbohydrate metabolism
B3W7V2	LCABL_05010	Fructose-bisphosphate aldolase	*fba*	Cytoplasmic	Yes			0.64	Carbohydrate metabolism

^a^ Proteins that are described in the literature as moonlighting proteins.

^b^ Ratio of protein abundance between mutant *srtA2* and *L*. *casei* wt, as determined by three different methods (see Material and Methods).

^c^ Detected in wt strain but not detected in *srtA2* mutant.

^d^ Detected in the *srtA2* mutant but not detected in the wt strain.

Two proteins containing the LPXTG motif were identified. Internalin J (InlJ) was found to be more abundant in the BL23 *srtA2* surfaceome, whereas Beta-N-acetylglucosaminidase (BnaG), was more abundant in BL23 wt.

Several proteins other than CWA proteins exhibited differential abundance in the cell surface proteome in BL23 *srtA2* and are involved in cell wall synthesis or degradation, exopolysaccharide biosynthesis, as well as in cell division processes. A greater abundance was observed in BL23 *srtA2* for the bifunctional dimerization/transpeptidase penicillin-binding protein 2B (Pbp2B2), UDP-N-acetylmuramyl-tripeptide synthetase (MurE), two proteins involved in exopolysaccharide biosynthesis (RmlB and Wzr), the septation ring formation regulator EzrA, and the signal recognition particle-docking protein (FtsY). On the contrary, a greater abundance was observed in the wt strain for N-acetylmuramoyl-L-alanine amidase, the cell-division proteins DivIVA and FtsX, and four proteins involved in exopolysaccharide biosynthesis (Wzb, RmlC, Wze and Wzd).

In addition, three proteins known as moonlighting proteins were found to be less abundant at the cell surface of BL23 *srtA2*. They included the fructose bisphosphate aldolase and two glyceraldehyde 3-phosphate dehydrogenases. Of note, the N-acetylmuramoyl-L-alanine amidase mentioned above is also known as a moonlighting protein [33].

Finally, another set of differentially abundant proteins included proteins involved in the interaction with the host cell and in the fitness of bacteria within their host. This group is comprised of proteins involved in stress response. Both thioredoxin reductase and thiol peroxidase, involved in oxidative stress response, were more abundant in BL23 *srtA2*, as were the ClpB chaperone, ClpC and ClpP protease. On the contrary, the glutathione peroxidase and the GrpE chaperone were more abundant in BL23 wt. In addition, three proteins involved in glycogen biosynthesis, GlgB, GlgC and GlgP, were also more abundant in the surfaceome of BL23 wt.

### *L*. *casei srtA2* has a thinner cell wall

**Fig 6:**
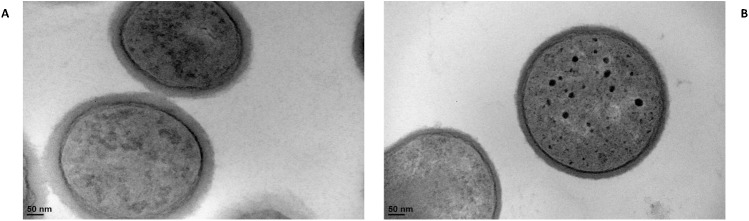
*L*. *casei* BL23 *srtA2* displayed a lower cell wall thickness compared to the wt strain. *L*. *casei* wt (A) and *srtA2* mutant (B) strains were grown in UF milk medium for 48 h and observed by transmission electron microscopy.

The impact of *srtA2* disruption on *L*. *casei* cell morphology was investigated using TEM on BL23 grown on UF milk medium until the stationary phase (48 hours). Although no alteration of the cell morphology (e.g., rod shape and length) was observed, a significant reduction (Student’s t-test; pval = 3.0 x 10^-15^) of the cell wall thickness was measured in BL23 *srtA2* compared to the BL23 wt control (Fig 6). Cell wall thickness was found to be 26.5 +/- 4.6 nm and 16.0 +/- 2.6 nm in *L*. *casei* BL23 wt and *srtA2* strains, respectively.

### *L*. *casei srtA2* presents a greater auto-aggregation ability

**Fig 7:**
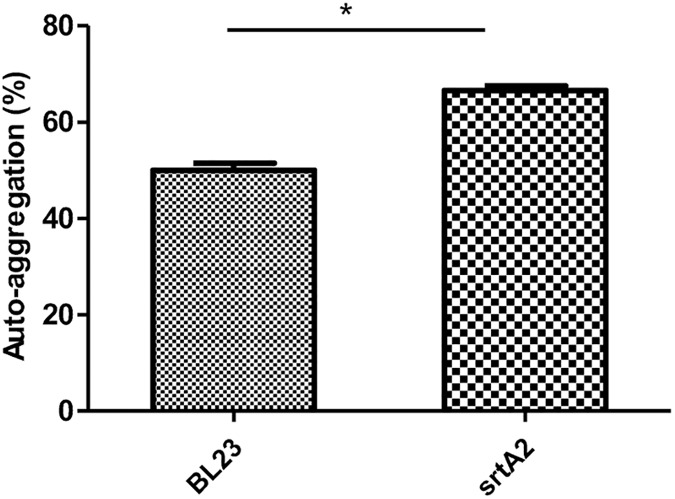
Auto-aggregation capacities of *L*. *casei* BL23 wt and *srtA2* strains. Strains were grown in UF milk medium for 48 h at 37°C. Auto-aggregation was evaluated by spectrophotometry (600 nm) and expressed as the auto-aggregation percentage. Cell suspension OD after growth (48 h) and homogenization was used as a reference (100%). Data are presented as means ± standard deviations. Each experiment was done in triplicate, and differences between groups were compared using Student’s t-test. *: P < 0.05.

The auto-aggregation capacity of *L*. *casei* BL23 wt and *srtA2* strains was assessed after a growth of 48 h in UF milk medium (stationary phase). The auto-aggregation rate of BL23 *srtA2* was slightly but significantly higher compared to that of the wt control, as illustrated by auto-aggregation rates of 50% and 66.67% in *L*. *casei* BL23 wt and *srtA2* strains, respectively (Fig 7). The total bacterial population counts were similar in both strains.

### *L*. *casei bnaG* retained its ability to inhibit *S*. *aureus* internalization

**Fig 8:**
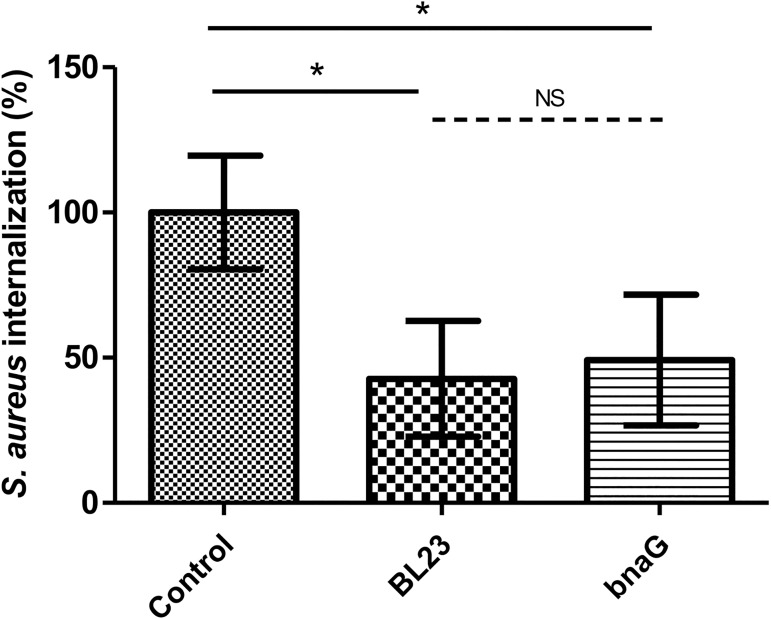
Impact of *L*. *casei* BL380 (BL23 *bnaG*) on *S*. *aureus* internalization into bMEC. Internalization rates of *S*. *aureus* N305 after 2 h of interaction with bMEC and co-incubation with *L*. *casei* BL23 and *L*. *casei* BL380 (*bnaG*) at an MOI of 2,000:1. *S*. *aureus* was used at an MOI of 100:1. The internalization assay of *S*. *aureus* alone was used as a reference. Internalization rates were then defined as the internalized *S*. *aureus* population in the presence of the different *L*. *casei* strains relative to the internalized *S*. *aureus* population of the reference experiment. Data are presented as means ± standard deviations. Each experiment was done in triplicate, and differences between groups were compared using one-way ANOVA with Bonferroni's Multiple Comparison Test. *: P < 0.05.

One of the LPXTG motif containing proteins whose abundance was higher in *L*. *casei* BL23 wt compared to *srtA2* mutant strain was the Beta-N-acetylglucosaminidase BnaG. In order to determine BnaG contribution to *L*. *casei* BL23 ability to inhibit *S*. *aureus* internalization into bMEC, a *bnaG* mutant strain was used in competition assays. *L*. *casei* BL23 *bnaG* retained its capacity to inhibit *S*. *aureus* internalization, at a level similar to the one obtained with BL23 wt strain (Fig 8).

## Discussion

In this work, we investigated the involvement of cell surface components in the capacity of *L*. *casei* BL23 to inhibit *S*. *aureus* internalization into bMEC. We first focused on FbpA as one of the main mechanisms involved in *S*. *aureus* internalization relies on the interaction between *S*. *aureus* fibronectin-binding protein and integrin α5 β1 via fibronectin bridging [17, 18]. However, we found that *L*. *casei fbpA* mutant strain inhibited *S*. *aureus* internalization at a level similar to that of the wt strain, suggesting that FbpA was not involved in inhibition mechanisms. Alternatively, we could not exclude the possibility that other BL23 proteins exhibiting fibronectin-binding activities contributed to this competition or that inhibition rely on several mechanisms and bacterial determinants that can compensate each other. It is interesting to note that the contribution of a fibronectin-binding protein (FnbA) to the inhibition of *S*. *aureus* adhesion to bMEC has been demonstrated for another LAB species, namely *Weissella cibaria* [34]. Such apparent discrepancies more likely point out the species-dependence or even strain dependence of such property.

We then focused on the *L*. *casei* sortase genes. Sortases specifically ensure the anchoring of CWA proteins in the cell wall of Gram-positive bacteria. The disruption of sortase genes thus directly affects the bacterial surfaceome in terms of CWA protein display and also indirectly affects the surfaceome through other changes in surface components [35]. The effect of sortase gene disruption in *L*. *casei* BL23 was thus evaluated with regard to surface properties, colonization capacities and inhibition potential against *S*. *aureus* bMEC invasion. All sortase mutant strains resulted in a reduced *L*. *casei* BL23 ability to inhibit *S*. *aureus* internalization into bMEC. The most pronounced effect was observed with the BL23 *srtA2* mutant. A significant release of inhibition was observed with a second *srtA2* mutant strain (*ΔsrtA2*), confirming that the loss of inhibition most likely relied on *srtA2* inactivation than on a secondary mutation. The internalization capacity of *L*. *casei* BL23 *srtA2* alone into bMEC was also reduced. We thus focused on this BL23 *srtA2* mutant to investigate changes in surface properties that may contribute to the loss of inhibition, by combining i) surface proteome analysis, ii) TEM observations of bacterial cell shape and of *L*. *casei*-infected bMEC, and iii) phenotypic characterizations. The *srtA2* mutation resulted in several changes at the bacterial surface, which, for some of them or all together, probably contribute to the loss of inhibition properties. These changes affect cell surface-exposed proteins beyond the direct substrates of sortases (i.e., proteins containing an LPXTG motif and a C-terminal cell wall anchor structure), moonlighting protein abundance, and they include modifications in the cell wall thickness and biosynthesis as well as in the oxidative stress response. Modification of auto-aggregative capacities of *L*. *casei* BL23 *srtA2* points out significant changes at the bacterial cell surface that may modify interaction with bMEC and thus competition with *S*. *aureus*.

In both pathogenic and probiotic bacteria, some surface proteins are important molecules for colonization and persistence in the ecosystem, as well as for cross-talk with the host cells and with the immune system [36]. Among these surface proteins, some CWA proteins have been described as important proteins since they promote the adhesion or invasion process in eukaryotic cells [37]. In this study, only two proteins bearing the LPXTG motif were found to be differentially abundant in the BL23 wt and *srtA2* surfaceome. Internalin J (InlJ) was more abundant in *L*. *casei srtA2*, whereas Beta-N-acetylglucosaminidase (BnaG) was more abundant in *L*. *casei* BL23 wt. In *Listeria monocytogenes*, InlJ reportedly contributes to virulence but its precise function is not known and it is not directly involved in cell invasion, contrarily to Internalin A and B [38, 39]. Internalin-like proteins have been described in other bacteria, including food-grade bacteria like *L*. *casei* or *Propionibacterium freudenreichii* [25], but their biological function remains unclear [39, 40]. Of note, *L*. *casei* InlJ does not contain an N-terminal leucine-rich repeat (LRR) domain, which is characteristic of internalin family members [39]. Whether InlJ in *L*. *casei* BL23 contributes to cell invasion remains to be determined. BnaG, the second LPXTG motif-containing protein identified here, harbors a GH20_DspB_LnbB-like domain (glycosyl hydrolase family 20 (GH20) catalytic domain of dispersin B (DspB), lacto-N-biosidase (LnbB) and related proteins) [41, 42]. *L*. *casei* BL23 BnaG was recently characterized. It is an extracellular enzyme involved in the metabolism of lacto-N-triose [22], a compound found in human milk oligosaccharides as well as in the glycan moieties of glycoproteins. The GH20_DspB_LnbB-like catalytic domain is also found in dispersin B, a glycoside hydrolase that hydrolyzes the beta-1,6-linkages of PNAG (poly-beta-(1,6)-N-acetylglucosamine), a major component of the extracellular polysaccharide matrix. This polysaccharide is notably produced by several staphylococcal species, including *S*. *aureus* and *Staphylococcus epidermidis* [43]. It is considered as an important adhesin that facilitates adhesion to biomaterials [41, 44]. As BnaG was a promising candidate that could account for the *srtA2* mutant phenotype, we tested the impact of *bnaG* deletion on *L*. *casei* BL23 capacity to inhibit *S*. *aureus* internalization. However, *L*. *casei bnaG* mutant strain retained an inhibition capacity similar to *L*. *casei* BL23 wt. This indicates that either *bnaG* was not involved in the inhibition phenotype or that inhibition relied on several surface components, resulting in a limited effect of *bnaG* deletion.

Moonlighting proteins are a special class of multifunctional proteins, some of which have functions related to adhesion [45]. Here, three proteins, mainly known for their intracellular role in glycolysis and gluconeogenesis and that exhibit moonlighting functions in other bacteria [46], were identified with greater abundance in the BL23 wt strain. They include two glyceraldehyde-3-phosphate dehydrogenase (GAPDH) and a fructose-bisphosphate aldolase (FBA). GAPDH was found extracellularly and shown to bind to human colonic mucin in *Lactobacillus plantarum* LA 318. Likewise, in the group A *Streptococci*, GAPDH displayed multiple binding activities to fibronectin, lysozyme, myosin and actin [47, 48]. Likewise, FBA has been found to be surface-exposed in several bacteria, including pathogens such as *Neisseria meningitidis*, which causes meningitis and septicemia [49]. In this species, FBA was shown to contribute to the adhesion to human brain microvascular endothelial and HEp-2 cells.

An additional moonlighting protein, N-acetylmuramoyl-L-alanine amidase, was found in greater abundance in *L*. *casei* BL23 wt compared to BL23 *srtA2*. N-acetylmuramoyl-L-alanine amidase is an enzyme that cleaves the amide bond between N-acetylmuramoyl residues and L-amino acid residues in bacterial cell walls. This hydrolase was also identified in *Mycobacterium tuberculosis* as an adhesin capable of binding to fibronectin and laminin [50]. The lower relative abundance of all these moonlighting proteins in BL23 *srtA2* may contribute to its altered colonization properties and inhibition capacity.

In addition to surface proteins, other components of the bacterial surface, including peptidoglycan and exopolysaccharides, are known to interact with the host. Peptidoglycan plays an important role in protecting the bacterial structural integrity and allows the covalent or non-covalent anchoring of various structures such as teichoic acids, polysaccharides and proteins [51]. Peptidoglycan fragments, when released after degradation, can induce an immune response in the host cells [52]. Exopolysaccharides (EPS), including capsular polysaccharide, wall PS or secreted ones, have also been shown to be involved in interactions with the host and, notably, interactions with the immune system in both probiotic and pathogenic strains [51, 53]. Electron microscopy analysis showed that the cell wall was thinner in *L*. *casei* BL23 *srtA2*. When considering the other changes identified here in the BL23 *srtA2* surfaceome, this suggests a possible imbalance in the bacterial cell wall turnover and/or EPS biosynthesis. Three proteins involved in the cell wall synthesis process were differentially abundant on the cell surface in *L*. *casei* BL23 *srtA2* compared to the wt strain. These include the bifunctional dimerization/transpeptidase penicillin-binding protein 2B (Pbp2B2) and a UDP-N-acetylmuramyl-tripeptide synthetase (MurE) that were more abundant on the BL23 *srtA2* surface. On the contrary, an N-acetylmuramoyl-L-alanine amidase was more abundant in the wt surfaceome. This peptidoglycan hydrolase cleaves specific bonds in peptidoglycan, thus contributing to the insertion of newly synthesized peptidoglycan subunits and to the separation of daughter cells following division [36]. We did not microscopically observe changes in cell size or cell separation, but it can be noted that four proteins involved in cell separation were also differentially abundant on the cell surface on wt and *srtA2* mutant strains. In addition, several proteins encoded within the EPS biosynthesis gene cluster displayed modified abundance in the *srtA2* mutant compared to *L*. *casei* BL23 wt. They include two proteins involved in NDP-sugar (EPS-precursors) biosynthesis (RmlB, RmlC) and three proteins involved in polysaccharide-chain-length determination (Wzd, Wze, Wzb), which were less abundant in *L*. *casei* BL23 *srtA2* than in the wt strain, with the exception of RmlB [53, 54]. Wzr was only detected in the *L*. *casei* BL23 *srtA2* surfaceome. The *wzr* gene is organized in the opposite transcriptional sense and probably encodes a transcriptional regulator whose exact function is unknown. EPSs have been shown to influence intercellular interactions and adhesion to biotic and abiotic surfaces by contributing to the cell surface physicochemical properties and/or by hiding some of the surface-exposed proteins, including some adhesins [55]. EPSs thus play a role in the formation of microcolonies and biofilms. Inactivation of *wzb*, which is involved in polysaccharide-chain-length determination in *Lactobacillus rhamnosus* GG, resulted in altered biofilm formation capacities [56]. Whether modulations of peptidoglycan and /or EPS biosynthesis account for the thinner cell wall of *L*. *casei* BL23 *srtA2* remains to be determined. Similarly, the impact of these modifications on *L*. *casei* BL23 interactions with bMEC is subject to further investigation.

Interestingly, when observing internalized *L*. *casei* BL23 wt and *srtA2* strains by TEM, a higher number of degradation vesicles were observed in bMEC infected with the mutant strain, suggesting that the lower internalization rate may be due to a lower capacity to invade cells, combined with a lower capacity to survive intracellularly. This was supported by a lower resistance of BL23 *srtA2* to oxidative stress, one of the harmful mechanisms used by eukaryotic cells to reduce bacterial viability [57]. In agreement with an impact of the *srtA2* mutation on oxidative stress resistance, three oxidative stress-related proteins, thioredoxin reductase, thiol peroxidase and glutathione peroxidase, were found to be differentially abundant in BL23 wt and *srtA2* surfaceomes. Several additional proteins related to stress response, including chaperones and proteases, were also differentially abundant in BL23 wt and *srtA2* surfaceomes. ClpC was notably shown to contribute to the persistence capacity of *L*. *plantarum* WCFS1 in murine GIT [58]. Finally, three proteins involved in glycogen metabolism were more abundant in the surfaceome of the wt strain. Glycogen metabolism is associated with energy storage and various physiological functions, including colonization and persistence [59]. Alteration of stress response capacity and, consequently, of intracellular survival of *L*. *casei* BL23 *srtA2* may also result in modifications of its inhibitory capacities against bMEC invasion by *S*. *aureus*.

In conclusion, these results strongly support a major role, either direct or indirect, of SrtA2 in *L*. *casei* BL23 inhibition capacity with regard to *S*. *aureus* internalization. The analysis of *srtA2* disruption on BL23 showed that it resulted in pleiotropic effects, including several changes in the surface proteome, beyond the LPXTG substrates, and changes at the cell wall level. Altogether, these results provide numerous presumptions about the functions (cell wall biosynthesis, oxidative stress and adhesion) putatively involved in this inhibition. They therefore open avenues for continuing research into this phenomenon since the candidates identified here could be targets for further characterization of this promising *S*. *aureus*-*L*. *casei*-bMEC tripartite interaction.

## Supporting information

S1 Table *L*. *casei* strains used in this study. (DOCX)

S2 Table Protein abundance ratios at the cell surface between the *srtA2* mutant and *L*. *casei* BL23 wt determined by Peak Counting. (XLSX)

S3 Table Protein abundance ratios at the cell surface between the *srtA2* mutant and *L*. *casei* BL23 wt determined by Spectral Counting. (XLSX)

S4 Table Protein abundance ratios at the cell surface between the *srtA2* mutant and *L*. *casei* BL23 wt determined by Extracted Ion Chromatogram (XIC). (XLSX)
