# Supplementary material for: Contribution of sortase SrtA2 to Lactobacillus casei BL23 inhibition of Staphylococcus aureus internalization into bovine mammary epithelial cells
Source: PLoS One. 2017 Mar 21;12(3):e0174060. doi: 10.1371/journal.pone.0174060 (PMC5360332; doi:10.1371/journal.pone.0174060)
Supplement: S1 Table — (DOCX) [file pone.0174060.s001.docx]

| **Strain** | **Relevant characteristic or description** | **Reference** |
| --- | --- | --- |
| *L. casei* BL23 | Wild type (wt) | 21 |
| *L. casei* BL308 | BL23 fbpA::pRV300, eryR (hereafter referred to as *fbpA*) | 19 |
| *L. casei* BL341 | BL23 srtA1::pRV300 EryR (hereafter referred to as *srtA1*) | 20 |
| *L. casei* BL342 | BL23 srtA2::pRV300 EryR (hereafter referred to as *srtA2*) | 20 |
| *L. casei* BL343 | BL23 srtC1::pRV300 EryR (hereafter referred to as *srtC1*) | 20 |
| *L. casei* BL344 | BL23 srtC2::pRV300 EryR (hereafter referred to as *srtC2*) | 20 |
| *L. casei* BL345 | BL23 ΔsrtA2 srtA1::pUCm1 CmR (hereafter referred to as *srtA1 srtA2*) | 20 |
| *L. casei* BL346 | BL23 ΔsrtA2 (hereafter referred to as Δ*srtA2*) | 20 |
| *L. casei* BL380 | BL23 bnaG (frameshift at SphI site) (hereafter referred to as *bnaG*) | 22 |
